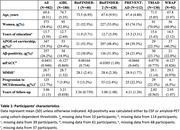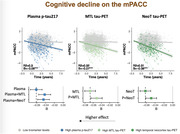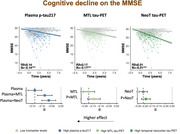# A head‐to‐head comparison between plasma p‐tau217 and tau‐PET for predicting future cognitive decline among cognitively unimpaired individuals

**DOI:** 10.1002/alz.090966

**Published:** 2025-01-09

**Authors:** Rik Ossenkoppele, Gemma Salvadó, Shorena Janelidze, Alexa Pichet Binette, Joseph Therriault, Erin M. Jonaitis, Sebastian Palmqvist, Niklas Mattsson‐Carlgren, Erik Stomrud, Pierrick Bourgeat, Vincent Dore, Colin L Masters, Sterling C. Johnson, Sylvia Villeneuve, Pedro Rosa‐Neto, Christopher C. Rowe, Oskar Hansson

**Affiliations:** ^1^ Amsterdam UMC location Vrije Universiteit Amsterdam, Alzheimer Center Amsterdam, Neurology, De Boelelaan 1117, Amsterdam, Noord‐Holland Netherlands; ^2^ Clinical Memory Research Unit, Lund University, Lund Sweden; ^3^ Clinical Memory Research Unit, Department of Clinical Sciences, Lund University, Lund Sweden; ^4^ Memory Clinic, Skåne University Hospital, Malmö Sweden; ^5^ Douglas Mental Health University Institute, Montreal, QC Canada; ^6^ Department of Medicine, University of Wisconsin‐Madison School of Medicine and Public Health, Madison, WI USA; ^7^ Clinical Memory Research Unit, Department of Clinical Sciences Malmö, Faculty of Medicine, Lund University, Lund Sweden; ^8^ The Australian e‐Health Research Centre, Commonwealth Scientific and Industrial Research Organisation, Brisbane, QLD Australia; ^9^ Department of Molecular Imaging, Austin Health, Melbourne, VIC Australia; ^10^ Florey Institute of Neuroscience and Mental Health, Parkville, VIC Australia; ^11^ University of Wisconsin‐Madison School of Medicine and Public Health, Madison, WI USA; ^12^ Douglas Mental Health Research Centre, Montreal, QC Canada; ^13^ Austin Health, Heidelberg, VIC Australia

## Abstract

**Background:**

An accurate prediction of Alzheimer’s disease (AD) progression is important for patient management and optimization of participant selection for trials. Here, we compared and combined plasma p‐tau217 and tau‐PET measures for predicting longitudinal cognitive decline and clinical progression in cognitively unimpaired participants.

**Method:**

We included 982 participants from six independent cohorts (AiBL, BioFINDER‐1, BioFINDER‐2, TRIAD, PREVENT‐AD and WRAP; Table 1) with available plasma p‐tau217 and tau‐PET measures (measured less than one‐year apart), being either amyloid‐positive or amyloid‐negative. Biomarker and cognitive data were z‐scored by cohort using cognitively unimpaired CSF/PET amyloid‐negative participants as reference for a unified analysis. We performed linear mixed models (for predicting cognitive decline on the mPACC and MMSE) and Cox‐proportional hazards models (to assess progression to MCI or dementia), testing among both amyloid‐negative and amyloid‐positive individuals. We entered baseline plasma p‐tau217, and tau‐PET uptake in the medial temporal lobe (MTL) or in the temporal neocortex individually, and also performed combined plasma/PET models. Age, sex, years of education and cohort were used as covariates.

**Result:**

All individual tau biomarkers significantly predicted cognitive decline for both mPACC (R^2^_p‐tau217_=0.27, R^2^_MTL‐tau_=0.31, R^2^_neotemporal‐tau_=0.28, Figure 1) and MMSE (R^2^_p‐tau217_=0.12, R^2^_MTL‐tau_=0.16, R^2^_neotemporal‐tau_=0.19, Figure 2). The best model for predicting mPACC change included plasma p‐tau217 and MTL tau‐uptake (R^2^=0.32, p_comparison_<0.001), while for MMSE change included plasma p‐tau217 and tau‐uptake in the temporal neocortex (R^2^=0.20, p_comparison_≤0.007). Progression to MCI or dementia was also best predicted when including both plasma p‐tau217 (HR[95%CI]=1.29[1.14,1.47], p<0.001) and MTL tau‐uptake (HR[95%CI]=1.39[1.25,1.54], p<0.001, c‐index=0.83). Analyses by individual cohorts showed similar trends.

**Conclusion:**

Our data suggest that plasma p‐tau217 is a suitable screening method for clinical trials in CU populations given its logistic advantages. In scenarios where a more refined prediction of cognitive decline is mandated, Tau‐PET (preferably in a combined algorithm with plasma p‐tau217) would be the methodology of choice.